# Prohibitin 1 tethers lipid membranes and regulates OPA1-mediated membrane fusion

**DOI:** 10.1016/j.jbc.2024.108076

**Published:** 2024-12-13

**Authors:** Tadato Ban, Kimiya Kuroda, Mitsuhiro Nishigori, Keisuke Yamashita, Keisuke Ohta, Takumi Koshiba

**Affiliations:** 1 Department of Protein Biochemistry, Institute of Life Science, Kurume University, Fukuoka, Japan; 2 Department of Chemistry, Faculty of Science, Fukuoka University, Fukuoka, Japan; 3 Advanced Imaging Research Center, Kurume University School of Medicine, Fukuoka, Japan

**Keywords:** coiled-coil, membrane fusion, mitochondria, prohibitin 1, proteoliposomes

## Abstract

Prohibitins (PHBs) are ubiquitously expressed proteins in the mitochondrial inner membrane (MIM) that provide membrane scaffolds for both mitochondrial proteins and phospholipids. Eukaryotic PHB complexes contain two highly homologous PHB subunits, PHB1 and PHB2, which are involved in various cellular processes, including metabolic control through the regulation of mitochondrial dynamics and integrity. Their mechanistic actions at the molecular level, however, particularly those of PHB1, remain poorly understood. To gain insight into the mechanistic actions of PHB1, we established an overexpression system for the full-length recombinant protein using silkworm larvae and characterized its biophysical properties *in vitro*. Using recombinant PHB1 proteoliposomes reconstituted into MIM-mimicking phospholipids, we found that PHB1 forms an oligomer *via* its carboxy-terminal coiled-coil region. A proline substitution into the PHB1 coiled-coil collapsed its well-ordered oligomeric state, and its destabilization correlated with mitochondrial morphologic defects. Negative-staining electron microscopy revealed that homotypic PHB1−PHB1 interactions *via* the coiled-coil also induced liposome tethering with remodeling of the lipid membrane structure. We clarified that PHB1 promotes membrane fusion mediated by optic atrophy 1 (OPA1), a key regulator of MIM fusion. Additionally, the presence of PHB1 reduces the dependency of lipids and OPA1 for completing the fusion process. Our *in vitro* study provides structural insight into how the mitochondrial scaffold plays a crucial role in regulating mitochondrial dynamics. Modulating the structure and/or function of PHB1 may offer new therapeutic potential, not only for mitochondrial dysfunction but also for other cell-related disorders.

Mitochondria are highly dynamic organelles that undergo continual cycles of homotypic fusion and fission, and maintenance of these equilibrium processes optimizes signal transduction and metabolism in eukaryotes (1, 2). Recent studies in animal models demonstrated that genetic ablation of individual components involved in mitochondrial dynamics impairs organ function and whole-body metabolism (3, 4, 5, 6, 7). Abnormal mitochondrial dynamics are also related to several human diseases, such as Charcot-Marie-Tooth disease type 2A (8), autosomal dominant optic atrophy (9), and a lethal developmental disorder (10). Recent studies further revealed that an imbalance in mitochondrial dynamics could potentially suppress innate immune responses to viral infection (11, 12, 13).

Mitochondrial fusion defects greatly enhance both the heterogeneity and dysfunction of the mitochondrial population (14). In mammals, the fusion process is precisely regulated by three conserved large guanosine triphosphatases (GTPases) of the dynamin superfamily, which coordinate two mitofusins (Mfn1 and Mfn2) localized at the mitochondrial outer membrane (MOM), and optic atrophy 1 (OPA1), which has 8 isoforms (long [L-] and short [S-] forms) resulting from transcriptional and post-translational processing through mitochondrial proteases localized in the mitochondrial inner membrane (MIM) (1). Prohibitins (PHBs), comprising two homologous subunits in eukaryotes (PHB1 and PHB2), are ubiquitously expressed and form hetero-oligomeric PHB complexes in the MIM, providing membrane scaffolds for both mitochondrial proteins and phospholipids (15, 16). The PHB complexes also participate in mitochondrial fusion coupling with many cellular signaling processes (16). PHB complexes are proposed to provide a functional platform that sequesters mitochondrial proteases from their substrate, OPA1, with the scaffolds regulating the proteolytic processing of OPA1 (specifically, the ratio of L-OPA1 to S-OPA1), which is essential for maintaining the fusion potency of the GTPases (15, 17, 18). In fact, *PHB2* gene ablation causes embryonic lethality in mice (17), providing insight into the requirement for PHB complexes to suppress mitochondrial fusion events through destabilizing fusogenic L-OPA1 by excess protease attack (*i*.*e*., accumulated S-OPA1 in the mitochondria). Although we are beginning to clarify the physiologic relevance of PHB complexes in mitochondrial biology, little is known about the mechanisms of action of PHB molecules in each process, particularly mitochondrial fusion.

In the present study, we focused on the mechanistic role of PHB1 and established an overexpression system for the intact recombinant protein using silkworm larvae to characterize its biophysical properties *in vitro*. We found that PHB1 forms a homotypic oligomeric state via a C-terminal coiled-coiled domain, thereby remodeling the membrane structure with a tethering lipid membrane and ultimately promoting OPA1-mediated membrane fusion. These structural insights help us to clarify the mechanistic roles of PHB complexes essential for mitochondrial fusion. Modulating the PHB1 structure and/or function may offer a new therapeutic target not only for mitochondrial dysfunction but also for other cell-related disorders outside mitochondria.

## Results

### Preparation of a recombinant PHB1 using silkworm larvae

**Figure 1:**
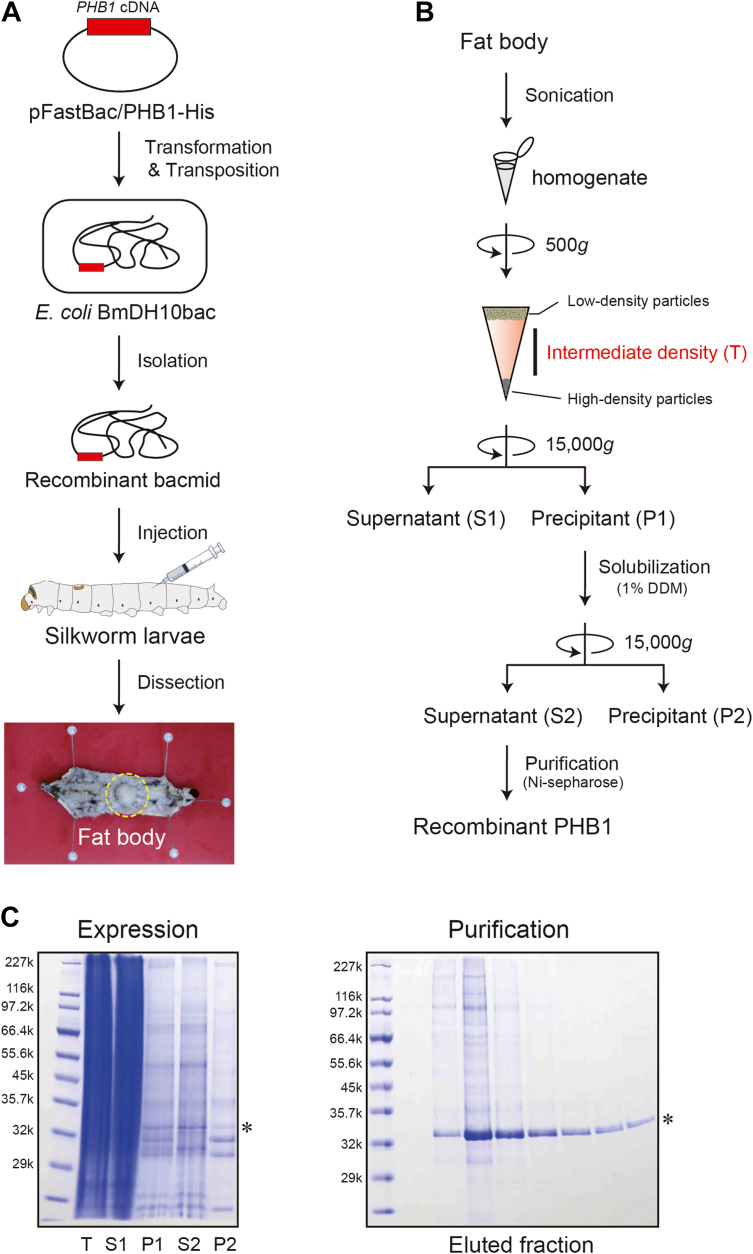
**Expression and purification of recombinant human PHB1 using silkworm larvae**. *A*, schematic of the silkworm expression system for the human PHB1 construct. A recombinant *Bombyx mori* nuclear polyhedrosis virus (BmNPV) DNA encoding PHB1 was injected into silkworm larvae. The recombinant protein was expressed in the fat body (*yellow dashed circle*) of the silkworm larvae. *B*, flow chart illustrating the purification steps of recombinant PHB1 expressed in the fat body from silkworm larvae. *C*, SDS-PAGE patterns of expressed recombinant PHB1. *Left* panel shows each loading pattern of the fractionated sample in (*B*). Lanes: (T) intermediate density; (S1) supernatant fraction 1; (P1) precipitation fraction 1; (S2) supernatant fraction 2; (P2) precipitation fraction 2. *Right* panel shows the purification pattern of the recombinant PHB1 using Ni Sepharose chromatography. The fat bodies from *n* = 30 silkworm larvae were used for the purification. Asterisks in both panels indicate the expressed recombinant protein.

To understand the precise mechanism underlying the involvement of PHB1 in the mitochondrial fusion process, we developed a technique for purifying a full-length recombinant PHB1 protein expressed in a eukaryotic system, silkworm larvae. A bacmid DNA encoding C-terminally histidine-tagged human *PHB1* was injected into silkworm larvae *in vivo* (Fig. 1*A*). Fat bodies storing the expressed recombinant protein were dissected, homogenized, and solubilized with detergent, followed by the purification of the extract using Ni-chelating resin (Fig. 1*B*). By rearing 30 transgenic silkworms, we obtained highly purified recombinant PHB1, which was eluded with imidazole (Fig.1*C*, asterisk). The final yield of the collected fractions was ∼0.3 mg per 30 silkworms as estimated by a Bradford protein assay.

### Biophysical properties of recombinant PHB1

**Figure 2:**
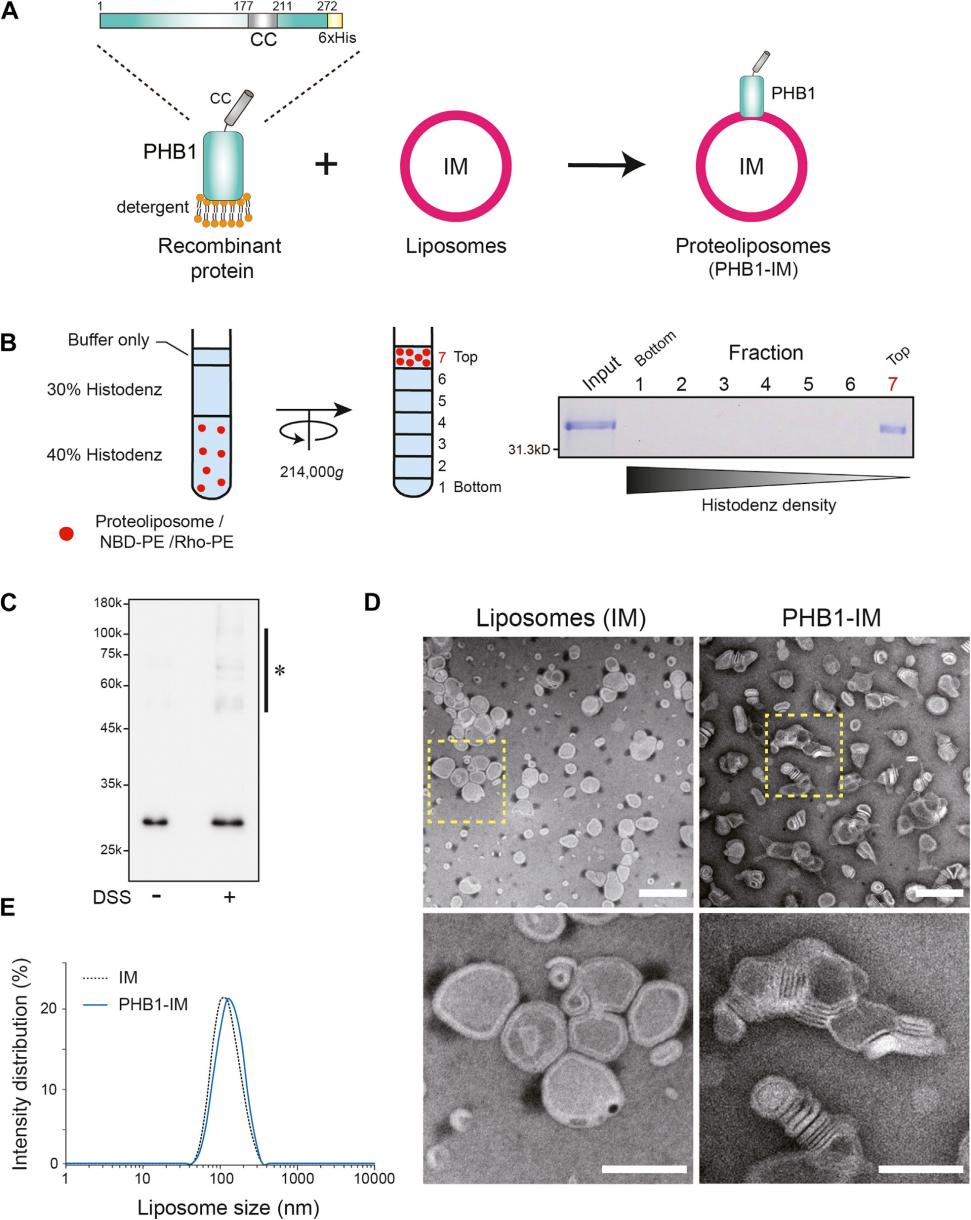
**Reconstitution of the recombinant PHB1 into MIM-mimicking liposomes**. *A*, recombinant PHB1 was integrated into either fluorescence-labeled or –non labeled MIM-mimicking liposomes (42% POPC, 25% POPE, 8% Soy-PI, and 25% CL) to produce proteoliposomes (PHB1-IM). *B*, liposome flotation assay. The reconstituted fluorescence-labeled PHB1-IM (*red* sphere) was layered over a linear gradient of Histodenz (*left*). After ultracentrifugation at 214,000*g*, 7 fractions were collected from the bottom and analyzed by SDS-PAGE (*right*). The top fraction (fraction 7) represents proteoliposomes showing a rhodamine fluorescence signal. *C*, a chemical cross-linking experiment of PHB1-IM using DSS reagent (final concentration of 1 mM) followed by Western blotting (α-His). Asterisk indicates homotypic oligomerized bands of PHB1. *D*, electron micrographic (negative staining) images of empty liposomes (IM) and PHB1 proteoliposomes (PHB1-IM). *Bottom* two images are magnifications of the area in the *yellow* dashed box in each top image. Scale bars, 200 nm (*top*) and 100 nm (*bottom*), respectively. *E*, particle sizes of liposomes reconstituted without (IM, *dotted* line) or with PHB1 (PHB1-IM, *blue* line) were determined by DLS measurement at 25^o^ C.

Because PHB1 is a membrane-bound protein localizing in the MIM (19), we next reconstituted proteoliposomes that contained our purified recombinant PHB1 within MIM-mimicking phospholipids (cardiolipin [CL]-enriched liposomes, IM) (Fig. 2*A*, denoted as PHB1-IM). Histodenz gradient-based liposome flotation analysis revealed that recombinant PHB1 is predominantly located in the top fraction (Fig. 2*B*, right). In this fraction, we could also visualize a rhodamine-labeled liposome marker (Rho-PE), suggesting that the recombinant PHB1 was properly targeted in the liposomes.

In a previous tissue culture experiment, we demonstrated that endogenous PHB1 undergoes homotypic oligomerization in mitochondria, which is essential for the function of the organelle (20). Notably, PHB1 also interacts with PHB2. We therefore investigated the biophysical properties of our reconstituted PHB1-IM, particularly its potential to form a complex. As expected, several oligomerized bands were detected in the presence of a crosslinker, disuccinimidyl suberate (DSS) (Fig. 2*C*, asterisk), suggesting that the recombinant PHB1 molecules tightly associated with themselves, consistent with endogenous proteins (20). More interestingly, our recombinant PHB1 exhibited a unique ability to influence membrane structures in the presence of MIM-mimicking phospholipids. Negative-staining electron microscopy (EM) of the PHB1-IM revealed that the observed PHB1-embedded liposomes were tightly packed and the flattened membranes folded into disk-like structures, reminiscent of the mitochondrial cristae morphology (Fig. 2*D*, right). In the absence of PHB1, some liposomes were tethered, but their membrane morphologies resembled normal bilayers with a single contact site in close proximity to each other (Fig. 2*D*, left). PHB1-dependent liposome tethering was further confirmed by dynamic light scattering (DLS) analysis, which revealed a shift in the size distribution of the proteoliposomes. A single peak observed in empty liposomes shifted toward a larger particle size when the PHB1-IM was analyzed (Fig. 2*E*, blue). In fact, these unique membrane structures were not observed in proteoliposomes when PHB1 was reconstituted within a MOM-mimicking phospholipid (*e*.*g*., lower composition of CL [∼4%], denoted as PHB1-OM) (Fig. S1, right) or in fusogenic L-OPA1-embedded proteoliposomes (21), indicating that the unique liposome remodeling property is a PHB1-specific phenotype. Taken together, these results suggest that PHB1 homotypic complexes are essential for remodeling membrane morphology, and the PHB1−PHB1 interaction might act *in trans* to tether opposing liposomes to change their structures.

### PHB1 coiled-coil mediates homotypic oligomerization and membrane deformation

**Figure 3:**
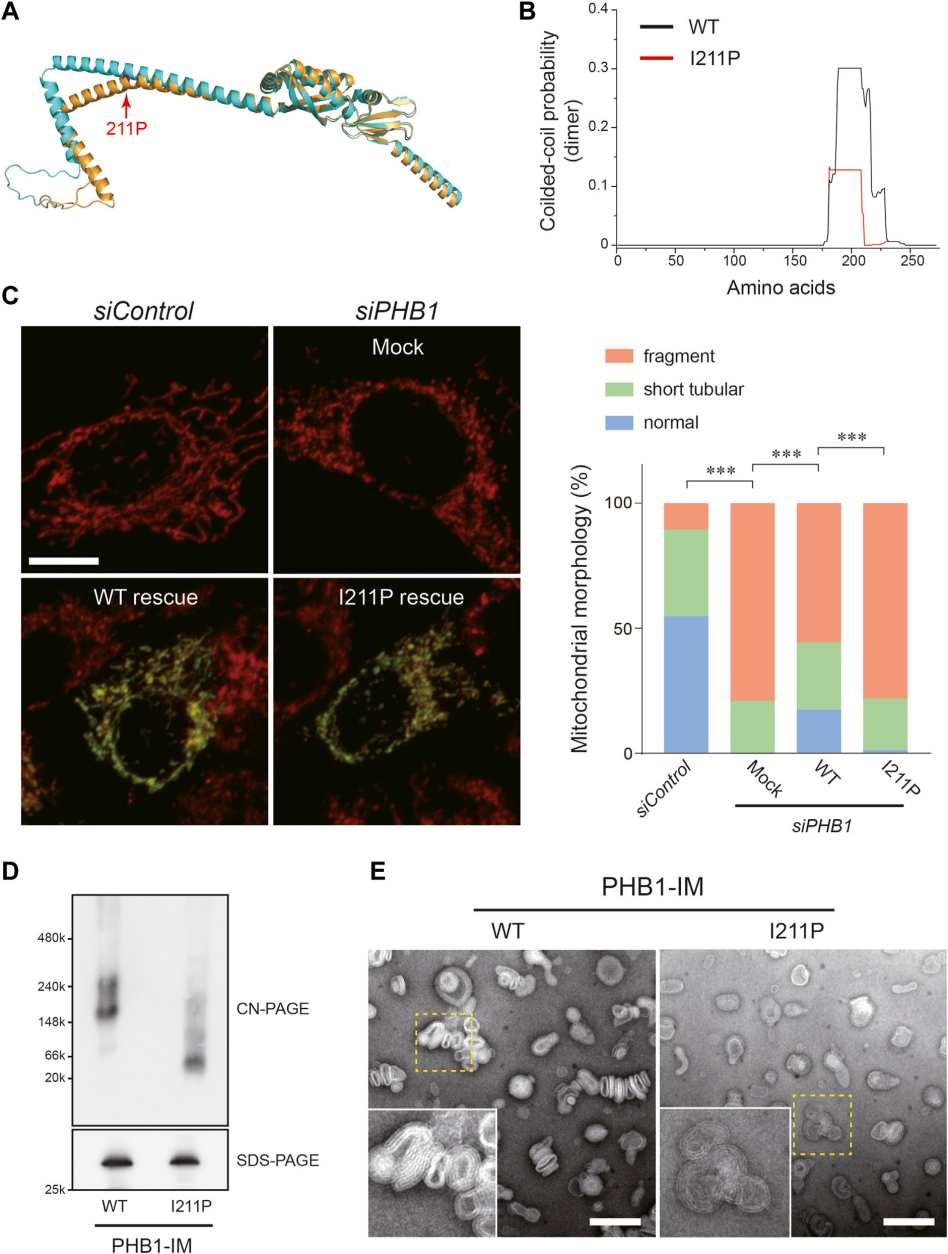
**Characteristics of the PHB1 coiled-coil essential for function**. *A*, a structural model of human PHB1 assigned by the AlphaFold2 program (40). Predicted structure of the I211P mutant (*orange*) is superimposed into the WT model (*cyan*) showing that the C-terminal α-helical fold is kinked by proline substitution. *B*, coiled-coil propensity predictions for the dimer model of WT (*black*) and I211P mutant (*red*) PHB1 generated using the COILS program (41). The WT PHB1 is predicted to have a higher coiled-coil probability compared with the I211P mutant. *C*, the siRNA-treated HeLa cells (against *PHB1*) were transfected with a Myc-tagged version of either a WT or I211P PHB1 expression plasmid, and stained with an antibody against the Myc epitope tag (*green*). Mitochondria in the same cells were also stained with an anti-COX-IV monoclonal antibody (*red*). We confirmed that mitochondrial morphology in the *siPHB1*-treated cells (Mock) was predominantly fragmented, whereas WT-rescued cells exhibited a tubular, networked mitochondrial morphology. The I211P rescued cells failed to recover the morphologic defect. Scale bar, 10 μm. Right, distribution of mitochondrial morphology in each *siControl* or *siPHB1*-treated cell. More than 300 cells were scored. ∗∗∗*p* < 0.001 (by chi-square test). *D*, PHB1 coiled-coil is involved in forming the PHB1 assembly. Reconstituted WT or I211P PHB1 proteoliposomes were solubilized in CN-PAGE lysis buffer containing 0.1% (w/v) digitonin, and analyzed by CN-PAGE and immunoblotted using the anti-PHB1 antibody (*top*). The abundance of the His-tagged proteins in each sample was confirmed by SDS-PAGE in parallel (*bottom*). *E*, negative-staining image of WT and I211P PHB1 proteoliposomes. Insets depict the magniﬁed images of each boxed area. Scale bar, 200 nm.

Our previous study indicated that another PHB1 isoform, PHB2, has a coiled-coil structure essential for its assembly formation (20). Because of the structural similarity between these two isoforms, we introduced a proline substitution into the coiled-coil region of PHB1, expecting that its folded structure would mildly collapse. *In silico* analysis confirmed that our proline substitution (I211P) into PHB1 kinked the C-terminal alpha-helix stretch (Fig. 3*A*, orange), significantly weakening the probability of the coiled-coil formation relative to that of the WT (Fig. 3*B*, red). To test whether the substitution actually affected the mitochondrial morphology in cells, we evaluated its role in the PHB1 coiled-coil structure using a *PHB1*-silencing approach. Knockdown of *PHB1* in HeLa cells *via* a small interfering RNA (siRNA) severely affected the mitochondrial morphologies and led to predominantly fragmented mitochondria in most cells (Fig. 3*C*, *siPHB1*_Mock), as observed in *PHB2*-deficient cells (17). The morphologic defects in the PHB1-attenuated cells were rescued by introducing the WT gene, but not by the I211P mutant (Figs. 3*C* & S2), suggesting that our predicted coiled-coil region in PHB1 was also functionally essential for mitochondrial dynamics.

We next performed *in vitro* assays to evaluate the biophysical properties of this mutant protein. Consistent with the functional assay in cells, the PHB1 homotypic oligomerization ability was partially lost when the proline substitution was introduced into the PHB1 coiled-coil, as revealed by clear native–polyacrylamide gel electrophoresis (CN-PAGE) (Fig. 3*D*) or a DSS cross-linking experiment (Fig. S3). Most critically, the ability of PHB1 to influence the liposome structures was well attenuated in the I211P proteoliposomes, with negative-staining EM showing an absence of the efficiently flattened membranes observed in the WT (Fig. 3*E*). The particle size distribution of the I211P mutant, which was shifted back toward the smaller size (Fig. S4, red), also supported the essential role of the PHB1 coiled-coil region for liposome tethering. Thus, these comparative *in vitro* and *vivo* studies highlighted the critical involvement of the PHB1 coiled-coil in mitochondrial dynamics by modulating the mitochondrial membrane architecture.

**Figure 4:**
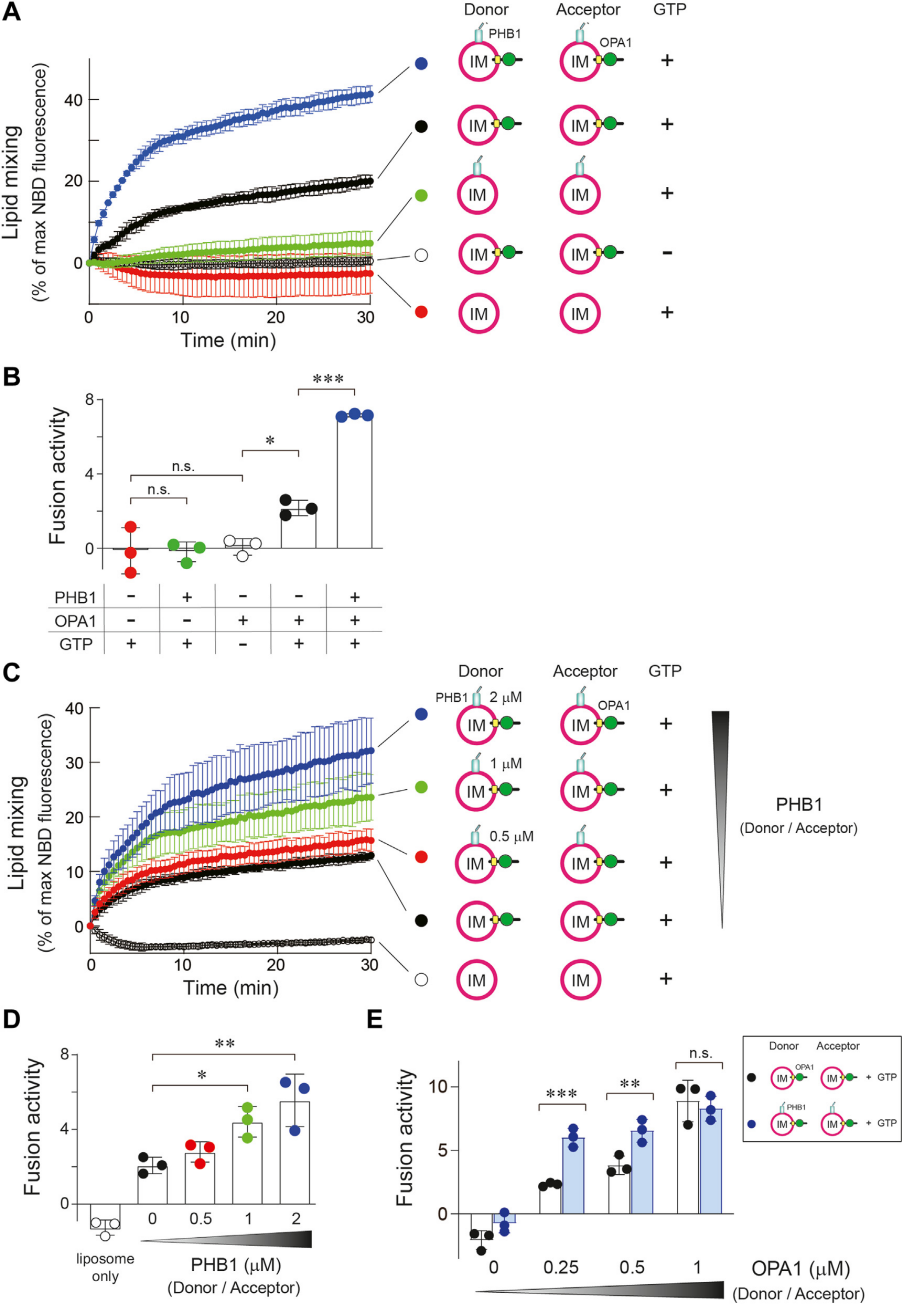
**OPA1-mediated liposome fusion assay.***A*, membrane fusion between each proteoliposome containing 0.5 μM L-OPA1 (*black* and *white*), 2 μM PHB1 (*green*), or both (*blue*) in the presence of GTP and MgCl_2_. Reduced fluorescence in the negative control (*red*) is due to photobleaching during measurement. *N* = 3 independent measurements. *B*, fusion activity was calculated from each trace from (*A*). All data shown represent mean values ± SD (*n* = 3 experiments). n.s., not significant, ∗*p* < 0.05, and ∗∗∗*p* < 0.001 (Tukey's multiple comparison test), respectively. *C*, similar to (*A*), except that the effect of increasing the amount of PHB1 (0.5, 1, and 2 μM) was evaluated by OPA1-mediated membrane fusion. In the experiment, 0.5 μM L-OPA1 with the same concentration of GTP and MgCl_2_ was used (*n* = 3 independent measurements). *D*, fusion activity was calculated from each trace in (C). All data shown represent mean values ± SD (*n* = 3 experiments). ∗*p* < 0.05, and ∗∗*p* < 0.01 (Tukey's multiple comparison test), respectively. *E*, the presence of PHB1 alters the concentration dependence of OPA1 for successful liposome fusion. The loss of fusion activity due to the decreased amount of L-OPA1 (0, 0.25, 0.5, and 1 μM, *black*) was complemented by PHB1 (1 μM, *blue*). All data shown represent mean values ± SD (*n* = 3 experiments). n.s., not significant, ∗∗*p* < 0.01, and ∗∗∗*p* < 0.001 (Bonferroni's multiple comparison test), respectively.

### PHB1 is involved in OPA1-mediated membrane fusion

The aforementioned experiments indicated the potency of the PHB1 coiled-coil in modulating membrane tethering/remodeling as well as mitochondrial morphology. To gain deeper insight into the mechanistic relevance of the PHB1 coiled-coil involvement in the mitochondrial fusion process, we used an OPA1-dependent membrane fusion assay (21) with an established fluorescence resonance energy transfer (FRET)-based *in vitro* membrane fusion method (22). When L-OPA1 (note that variant 1 was used in this assay) was reconstituted into both the donor and acceptor sides of MIM-mimic liposomes (*i*.*e*., OPA1-IM), a sufficient level of liposome fusion was observed in a GTP-dependent manner (Fig. 4*A*, black *versus* white), as previously described (21). In this FRET assay, we confirmed that PHB1 itself did not exhibit any FRET signal (green), but it demonstrated a greater potential to promote OPA1-mediated membrane fusion (PHB1/OPA1-IM, blue *versus* black). When the fusion activity of each trace was estimated by their initial time points (the first 2 min), the PHB1-containing OPA1-IM exhibited a highly pronounced FRET signal (Fig. 4*B*), indicating that the fusion activity was largely enhanced (∼3-fold) by PHB1.

To verify that the observed effect was truly due to the presence of PHB1 in the liposomes, we further analyzed OPA1-mediated membrane fusion by changing the amount of PHB1 (or changing the PHB1:OPA1 ratio) in the reconstituted proteoliposomes. First, the fusion events were accelerated by increasing the abundance of PHB1 in a dose-dependent manner (Fig. 4, *C* and *D*). Second, the presence of PHB1 definitely improved the fusion activities, especially when the OPA1 level was lower (Fig. 4*E*, < 1 μM). A higher abundance of OPA1 was sufficient to trigger membrane fusion on its own, but when OPA1 levels were reduced, fusion activity was effectively restored in the presence of PHB1 (Fig. 4*E*, blue). This suggests that PHB1 enhances the OPA1-mediated membrane fusion process.

#### Role of the PHB1 coiled-coil in OPA1-mediated membrane fusion

**Figure 5:**
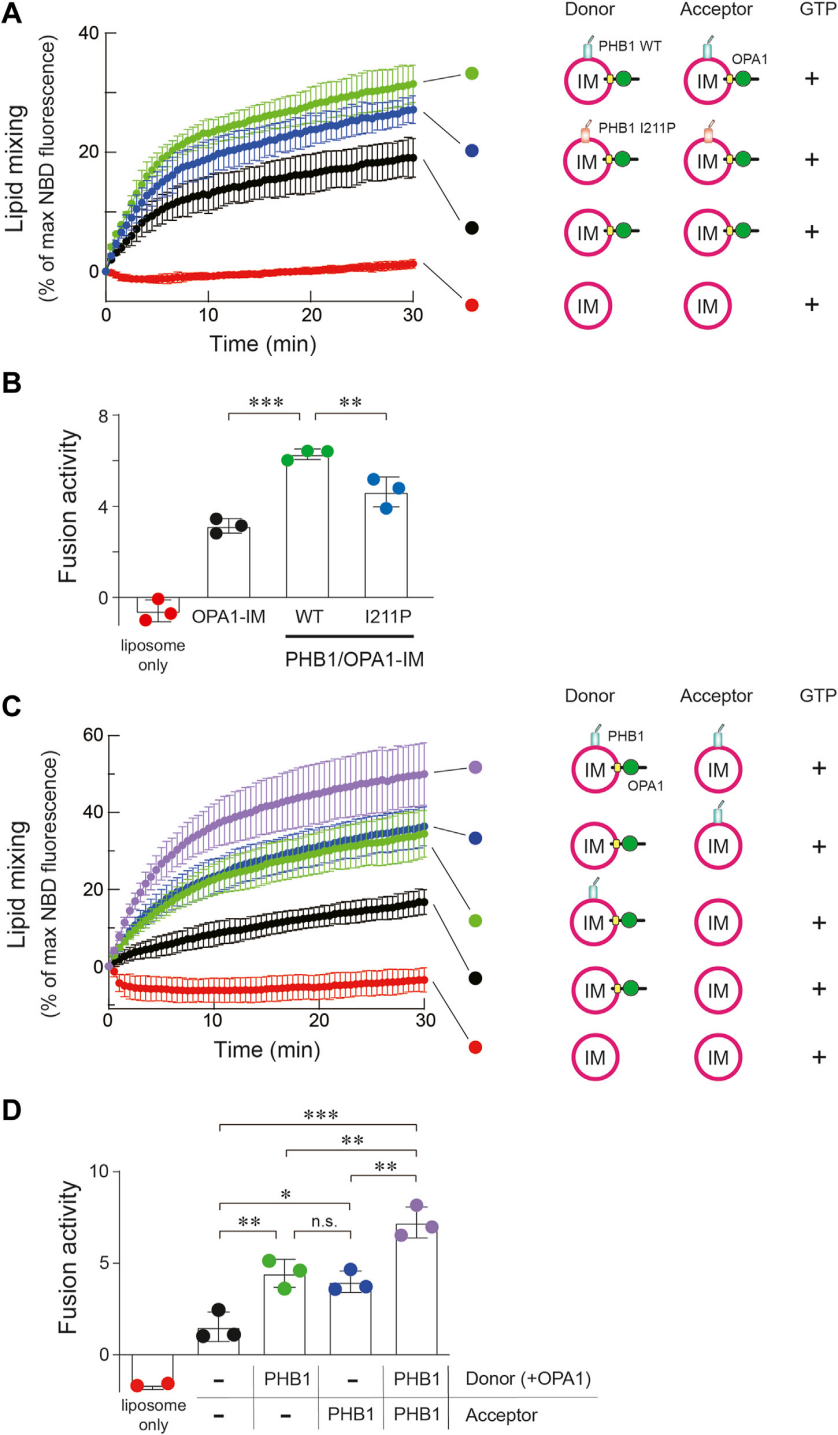
**Role of the PHB1 in OPA1-mediated membrane fusion**. *A*, The OPA1-mediated membrane fusion reactions were measured in the presence of either 1 μM WT (*green*) or I211P PHB1 (*blue*) (*n* = 3 independent measurements). Reduced fluorescence in the negative control (*red*) would be caused by photobleaching during measurement. In the experiment, 0.5 μM of L-OPA1 was used. *B*, fusion activity was calculated from each trace from (*A*). All data shown represent mean values ± SD (*n* = 3 experiments). ∗∗*p* < 0.01 and ∗∗∗*p* < 0.001 (Tukey's multiple comparison test), respectively. *C*, different combinations (donor side of 0.5 μM L-OPA1 without or with 1 μM PHB1) of the OPA1-mediated membrane fusion reaction were measured as in the case of (*A*). *N* = 3 independent measurements. *D*, fusion activity was calculated from each trace in (*C*). All data shown represent mean values ± SD (*n* = 3 experiments). n.s., not significant, ∗*p* < 0.05, ∗∗*p* < 0.01, and ∗∗∗*p* < 0.001 (Tukey's multiple comparison test), respectively.

We next investigated whether the PHB1 coiled-coil was involved in this membrane fusion by preparing reconstituted OPA1 proteoliposomes embedded with the PHB1 mutant (PHB1^I211P^/OPA1-IM). As expected, the mutant proteoliposomes were partially involved in the OPA1-mediated membrane fusion, but the effect was significantly smaller compared with that of WT PHB1 (Fig. 5, *A* and *B*). To clarify whether interactions between PHB1 and OPA1 in the liposomes are important for controlling membrane fusion, we assessed the level of OPA1 oligomerization induced by PHB1. Intriguingly, cross-linkage products of OPA1 were increased by the presence of WT PHB1, but the effect was not observed in the mutant proteoliposomes (Fig. S5). Although these data confirmed that the C-terminal coiled-coil region of PHB1 plays an essential role in promoting OPA1-mediated membrane fusion, we cannot entirely rule out the possibility that PHB1 adopts an alternative conformation or facilitates marginal fusion through mechanisms unrelated to the coiled-coil effect (Fig. 5*B*, blue).

#### The presence of PHB1 reduces the lipid dependency for OPA1-mediated membrane fusion

We investigated whether PHB1 is required on both sides of the liposome membrane for controlling the membrane fusion. L-OPA1 on one side (donor) could be enough to mediate membrane fusion (Fig. 5, *C* and *D*, black) because a high amount of unstable lipid CL on the other side (acceptor) would react with the L-OPA1 *in trans*, leading to heterotypic membrane fusion (21). In fact, the presence of PHB1 on either side (*cis-* or *trans-*in relation to L-OPA1) showed a synergistic effect on the membrane fusion process (green and blue), with the highest fusion activity of L-OPA1 observed when PHB1 was present on both sides (purple).

**Figure 6:**
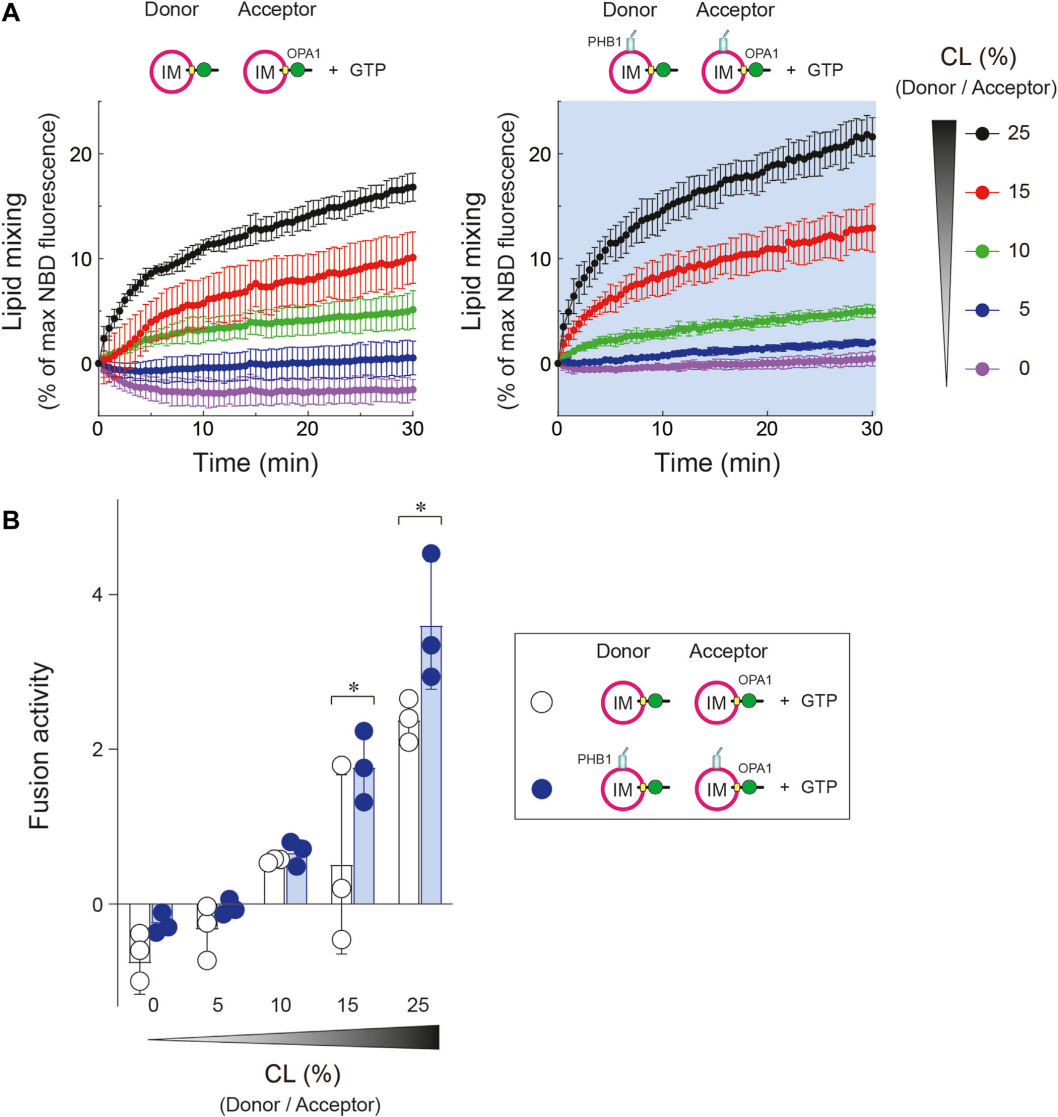
**PHB1 alters the CL-dependence for OPA1-mediated membrane fusion**. *A*, membrane fusion between donor and acceptor of OPA1/IM (0.5 μM L-OPA1) without (*left*) or with PHB1 (*right*, 1 μM) was measured by decreasing the amount of CL content (0, 5, 10, 15, and 25%) in the presence of GTP and MgCl_2_. *N* = 3 independent measurements. *B*, fusion activity was calculated from each trace in (*A*). All data shown represent mean values ± SD (*n* = 3 experiments). ∗*p* < 0.05 (Bonferroni's multiple comparison test).

We finally assessed whether the presence of PHB1 alters the CL-enriched dependence for OPA1-mediated membrane fusion. Normally, L-OPA1 requires a relatively high CL content (∼25%) to exhibit its fusion activity. In the presence of PHB1, however, this lipid dependence was reduced with significant fusion activity observed at a 15% CL content *in vitro* (Fig. 6, *A* and *B*). It is important to note that a CL content below 10% does not mimic the MIM lipid composition and more closely resembles the MOM. Therefore, PHB1 might not remodel the liposome structure at these lower CL levels, as observed in transmission EM (Fig. S1). Taken together, these results suggest that PHB1 on both sides of the liposome membrane enhances OPA1-mediated membrane fusion, likely due to liposome tethering and deformation induced by PHB1 homo-oligomerization.

## Discussion

PHBs are key mitochondrial scaffold molecules in the MIM (16) belonging to the stomatin, prohibitin, flotillin, and HflK/C (SPFH) family members, which also play scaffolding roles in microdomain formation by forming highly ordered oligomers in many species (23). Similarly, SPFH proteins not only comprise a rigid scaffold in subcellular membranes but also dynamic platforms due to their unique regulatory functions under certain circumstances (24). Many of these functions, however, at the molecular level remain unclear.

**Figure 7:**
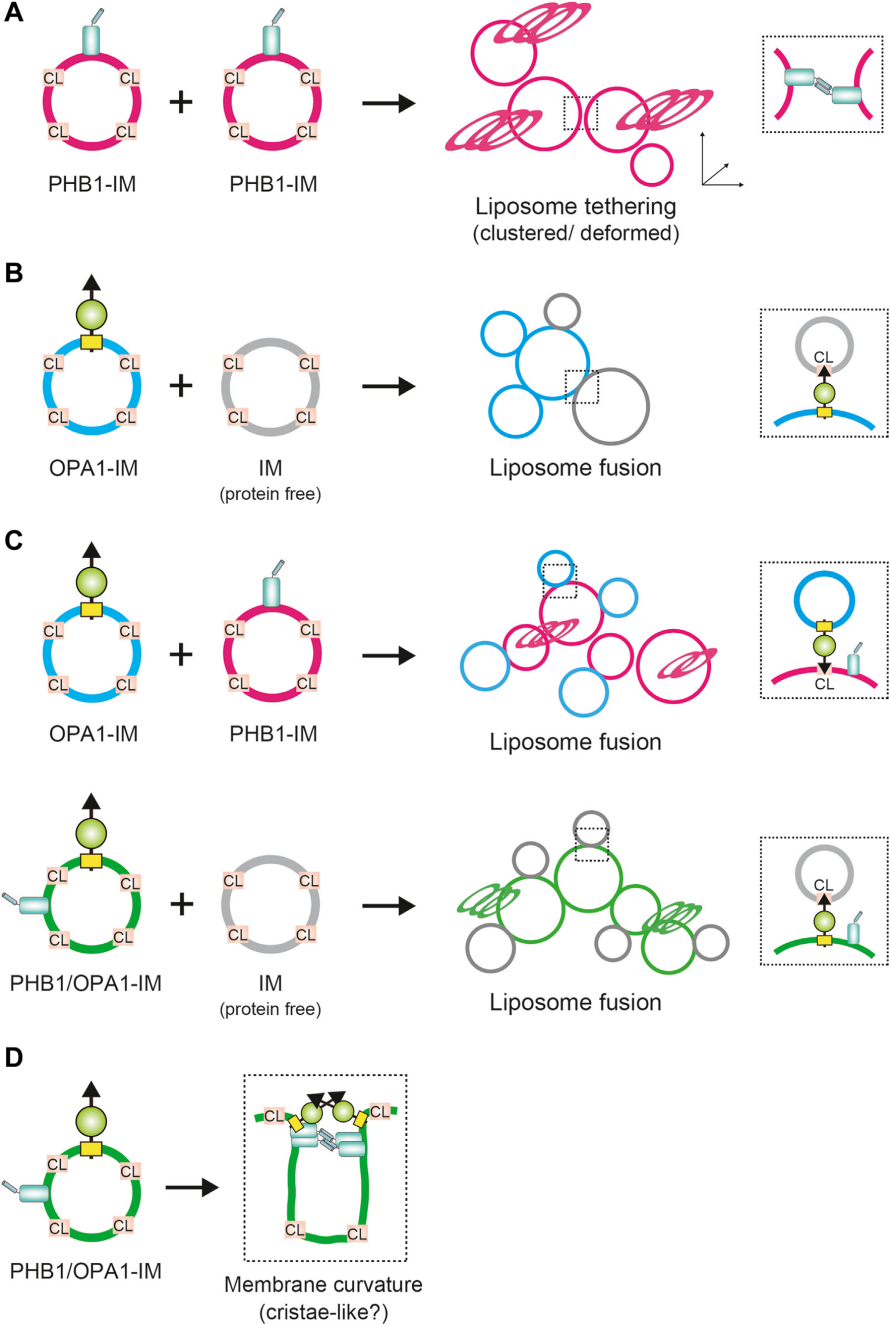
**Models of PHB1 involved in the OPA1-mediated membrane fusion**. *A*, model of a *trans*-PHB1 complex on liposomes. Formation of the PHB1 coiled-coil would tether two adjacent membranes and remodel the liposome structure to form clustering states and deformed membranes without any fusion event (inset). *B*, model for L-OPA1-mediated membrane fusion. L-OPA1 binds directly to an unstable phospholipid CL to tether the other side of the membrane (inset), and GTPase hydrolysis mediates further membrane fusion. *C*, similar models to (*B*), except that PHB1 exists on the opposite (*top*) or same (*bottom*) sides of the liposomes relative to L-OPA1. In both models, the resulting OPA1-mediated membrane fusion is due to the OPA1−CL interaction (inset), and the *trans*-PHB1 complex (clustered and deformed liposomes) enhances the probability of encountering L-OPA1 and CL in its vicinity. In addition, the presence of PHB1 would mitigate the CL and OPA1 dependency for completing the fusion event. *D*, model of a *cis*-PHB1 complex in liposomes. Homotypic interaction of PHB1 with an OPA1-pairing would lead to membrane curvature and ultimately form cristae-like structures (inset). See text for more details.

In the present study, we established a reconstitution system for PHB1 proteoliposomes using our recombinant protein expressed in silkworm larvae and showed that the recombinant protein had a high propensity to oligomerize *via* its coiled-coil domain at the C-terminus and initiate liposome deformation by tethering lipid membranes *in vitro*. We assume that these dynamic properties of PHB1 proteoliposomes, which attract protein and phospholipids from adjacent membranes, increase their binding frequency with L-OPA1 and the unstable phospholipid CL on the *trans* side of the liposome membrane. This interaction could trigger OPA1-mediated membrane fusion in the presence of GTP, facilitated by GTP hydrolysis (21, 25). Because PHB1 is not a fusogenic molecule, the proteoliposomes themselves do not exhibit any fusion events (Fig. 7*A*). Consistent with this assumption, our biochemical analysis indicated that PHB1 mediates liposome tethering and clustering, accompanied by membrane deformation, in a CL-dependent manner (Figs. 2*D* and S1), though membrane fusion events were not triggered (Fig. 4*A*). We hypothesize that homotypic PHB1−PHB1 interactions tether the adjacent membranes and allow the protein complexes to act *in trans* but without initiating the subsequent fusion events (Fig. 7*A*, inset). In general, most diverse membrane trafficking systems rely on *trans*-protein complexes, such as *trans*-SNAREpin (22) and *trans*-Mfn (26) complexes, to facilitate membrane tethering/docking before lipid bilayer fusion occurs. Although our PHB1 tethering model is based on a dimeric anti-parallel coiled-coil structure (20), we cannot exclude the possibility that PHB1 adopts alternative conformation states and may be induced into other folding patterns. Indeed, previous structural models demonstrated successive self-oligomerization of SPFH-domain proteins from an *Archaea*, even without a coiled-coil formation (27). Additionally, our crosslinking experiments combined with native PAGE suggest that weakly assembled states still exist in the PHB1 coiled-coil mutant (Figs. 3*D* & S3).

A fusogenic L-OPA1 on one side of a liposome membrane can potentially promote fusion with the opposite side, which contains a high CL content (∼25%), through a protein-lipid interaction (Fig. 7*B*, inset) (21). As mentioned above, PHB1 can remodel multiple liposomes, likely enhancing OPA1-mediated membrane fusion through its role as a tethering factor (Figs. 4 and 7*C*). Additionally, the presence of PHB1 ensures that OPA1 maintains its fusion activity, even when the CL content in liposomes is reduced (Fig. 6). Although our FRET data demonstrated that the presence of PHB1 only on one side of a liposome membrane is sufficient to contribute to OPA1-mediated membrane fusion (Fig. 5*C*), we could not determine its topologic relevance—whether the interaction between PHB1 and OPA1 occurs *in cis* or *trans*—or which configuration is more necessary for facilitating the fusion event. In this context, we propose another scenario in which the PHB1−PHB1 interaction partially acts *in cis* under certain circumstances (Fig. 7*D*). This is because our negative-staining EM images revealed that PHB1-embedded proteoliposomes were well-deformed and folded into disk-like structures (Fig. 2*D*) reminiscent of mitochondrial cristae. Previous ultrastructural analysis in *PHB2*-deficient cells revealed the defective morphogenesis of cristae in the absence of the PHB complexes (17, 28), underscoring the importance of PHBs in cristae maintenance. These observations raise a possibility that the *cis*-PHB1 complex likewise alters local membrane curvature, leading to an unstable membrane structure that ultimately stimulates OPA1-mediated membrane fusion (29).

Finally, it is important to note that several studies have pointed out the involvement of PHB in cell proliferation (30, 31) and viral entry (32, 33, 34), which might be distinct or indirect roles in mitochondrial pathways. Interestingly, these actions seem to be coupled with phosphorylation events in PHBs (30, 35, 36). Therefore, targeting PHBs could offer a promising avenue for drug design, potentially addressing not only mitochondrial dysfunction but also other cell-based disorders not involving the mitochondria.

## Experimental procedures

### Cloning and mutagenesis

The plasmid pcDNA3.1/PHB1-BirA-HA (20) was used as a template to amplify human PHB1 cDNA by polymerase chain reaction with each primer encoding 5′ *Not*I (including Kozak sequence) and 3′ *Hind*III (encoding 6 histidine residues just upstream at a stop codon) site. The amplified ∼850-base pair PHB1 fragment was ligated into the pFastBac1 plasmid (Life Technologies, Carlsbad, CA) to generate pFastBac/PHB1-6 × His. Proline substitution (I211P) into the pFastBac/PHB1-6 × His plasmid was introduced by site-direct mutagenesis according to the manufacturer’s protocol (New England Biolabs). Mammalian expression plasmids for human PHB1 and its variant were constructed by ligating each cDNA into the pcDNA3.1(−) vector (Invitrogen, Waltham, MA) that encoded a C-terminal 3 × Myc tag. All constructs used in the study were conﬁrmed by DNA sequencing (Applied Biosystems 3730xl Genetic Analyzer). X-tremeGENE HP DNA transfection reagent (Roche) was used for transfecting the expression plasmids into mammalian cells.

### Antibodies and reagents

Anti-*c*-myc (9E10, 1:1000) and anti-OPA1 (Cat# 612606, 1:1000) mouse monoclonal antibodies were purchased from Covance and BD Transduction Laboratories, respectively. Anti-PHB1 (ab75766, 1:1000) and anti-CoxIV (3E11, 1:1000) rabbit monoclonal antibodies were supplied from either Abcam or Cell Signaling Technology, and penta-His tag monoclonal antibody was obtained from Thermo Fisher Scientific. The Alexa Fluor 488 (1:1000) anti-mouse monoclonal and Alexa Fluor 568 (1:1000) anti-rabbit polyclonal antibodies were obtained from Molecular Probes/Invitrogen. GTP was purchased from FUJIFILM Wako Pure Chemical Corporation, and all other reagents used in the study were biochemical research grade.

### Protein expression and purification

PHB1 and L-OPA1 were expressed in silkworm fat bodies using the previously established *Bombyx mori* nucleopolyhedrovirus (BmNPV) bacmid-silkworm expression system (37). In brief, the constructed plasmids pFastBac/PHB1-6 × His and pFastBac HTb/6 × His-L-OPA1 (21) were each transformed into *Escherichia coli* BmDH10Bac, and recombinant BmNPV bacmid DNAs containing either PHB1 or L-OPA1 were purified using a Plasmid Maxiprep kit (QIAGEN). For expression of each recombinant protein, firth-instar larvae of silkworms were purchased from Ehime Sansyu and an aliquot of transfection mixture that contained each isolated BmNPV bacmid (1.5 μg) and 3 μl of DMRIE-C Transfection Reagent (Thermo Fisher Scientific) was injected into the dorsal side of each silkworm larva followed by rearing for 6 days in a 25° C incubator. At 6 days after infection, fat bodies from the silkworm were collected manually and homogenized in a phosphate buffer (pH 8.0) containing 50 mM sodium phosphate, 500 mM NaCl, 10% (w/v) glycerol, 1 mM dithiothreitol (DTT), 1 mM phenylmethylsulfonyl fluoride, and 0.5% sodium thiosulfate using a glass-Teflon potter homogenizer. The homogenized fat bodies from a total of 30 silkworms were disrupted by sonication and then centrifuged at 14,000*g* for 1h (at 4° C). Next, the pellets were resuspended into 40 ml of phosphate buffer containing 10 mM imidazole and 1% (w/v) *n*-dodecyl-β-D-maltoside and further incubated for 2 h at 4° C with gentle agitation. The suspended pellet was centrifuged at 14,000*g* for 30 min and the resulting supernatant containing the solubilized recombinant PHB1 and L-OPA1 fractions was collected.

For purification of the recombinant proteins, each sample was solubilized in detergent as described above and bound with 1 ml of Ni-Sepharose 6 fast flow beads (Cytiva) at 4° C overnight. The following day, the beads were transferred into a 1.0 × 5 cm chromatography column (Bio-Rad, Hercules, CA) and washed with 10 ml of RB buffer (20 mM Tris-HCl [pH7.4], 300 mM NaCl, 10% [w/v] glycerol, and 1 mM DTT) containing 20 mM imidazole and 0.2% (w/v) *n*-decyl-*β*-D-maltoside (DM) (Dojindo) to remove non-specific bound proteins from the beads. After washing once with 10 ml of the RB buffer containing 40 mM imidazole and 0.2% DM, bound proteins were eluted from Ni-Sepharose 6 beads using RB buffer containing 250 mM imidazole and 0.2% DM. Each eluted fraction was analyzed by sodium dodecyl sulfate (SDS)-PAGE and their concentrations were determined using a Bradford protein assay kit (Nacalai Tesque). Finally, the purified proteins were flash-frozen in liquid nitrogen and stored at −80° C.

### Preparation of proteoliposomes

Liposomes containing recombinant PHB1 and/or L-OPA1 were prepared by the detergent-dialysis method as described previously (21, 37) with slight modifications. Next, non-fluorescent lipids were used to prepare liposomes for the study; 1-palmitoyl-2-oleoyl-sn-glycero-3-phosphocholine (POPC), 1-palmitoyl-2-oleoyl-sn-glycero-3-phosphoethanolamine (POPE), L-α-phosphatidylinositol (Soy-PI), and 1′,3′-bis[1,2-dioleoyl-sn-glycero-3-phospho]-glycerol (CL), all purchased from Avanti Polar Lipids. Fluorescent-labeled lipids were obtained from Molecular Probes (Eugene, OR) and covalently labeled with *N*-(7-nitrobenz-2-oxa-1,3-diazol-4-yl)-1,2-dihexadecanoyl-sn-glycero-3-phosphoethanolamine (NBD-PE) and lissamine-rhodamine B 1,2-dihexadecanoyl-sn-glycero-3-phosphoethanolamine (Rho-PE). Based on the lipid composition of the MIM (38), we mimicked the lipid composition and used a ratio (percent molar) of POPC: POPE: Soy-PI: CL (42: 25: 8: 25) in the study (Of note, we used a lipid composition of POPC: POPE: Soy-PI: CL [42: 25: 9: 4] for MOM-mimicking liposomes). For the preparation of the fluorescence-labeled MIM liposomes, 3% of POPE was replaced with 1.5% NBD-PE and 1.5% Rho-PE. Their indicated mixed ratio of lipids was dissolved in chloroform solution and the solvent was dried under N_2_ gas. The dried lipid film was further dried under vacuum for 30 min and dissolved in RB buffer containing 5% (w/v) *n*-octanoyl-*N*-methyl-D-glucamine (MEGA-8, Dojindo) at a final concentration of 8 mM. Proteoliposomes were prepared by adding recombinant PHB1 at a concentration of 0.5 to 2 μM and/or L-OPA1 at a concentration of 0.25 to 1 μM with a detergent-lipid mixture containing 1.25% MEGA-8, 0.15% DM, and 2 mM lipids, and that incubating for 2 h at 4° C with gentle agitation. To remove excess detergent reagent and protein-empty forms of liposomes, the above protein-lipid mixtures were placed in a dialysis button (Hampton Research) and dialyzed against the RB buffer at 4° C with gentle agitation. The size distribution of the prepared liposomes and proteoliposomes was determined by DLS analysis using an nanoSAQLA instrument (Otsuka Electronics Co., Ltd) at 25° C.

### Membrane flotation assay

For the flotation assay (39), 750 μl of proteoliposomes was mixed with an equal volume of 80% (w/v) Histodenz (Sigma-Aldrich) in RB buffer. The sample was transferred into an 11 × 34 mm ultra-clear tube (Beckman Instruments) and layered with 30% (w/v) of Histodenz to a final volume of 2.2 ml. After adding 200 μl of RB buffer, the sample was centrifuged at 214,000*g* for 2 h in a TLS-55 rotor (Beckman Instruments). Each 300-μL fraction was collected from the bottom of the tube and their fractions were analyzed by CBB staining to confirm protein detection.

### Clear native (CN)-PAGE

Prepared proteoliposomes were lysed in RB300 buffer containing 20 mM Tris-HCl (pH 7.4), 300 mM NaCl, 1 mM DTT, 10% (v/v) glycerol, and 0.1% (w/v) digitonin on ice for 30 min. The clarified supernatant was supplemented with 0.01% (w/v) Ponceau-S, separated on 3 to 14% u-PAGEL (Atto), and immunoblotted with the anti-PHB1 antibody.

### Crosslinking assay

To analyze PHB1 oligomerization (or the PHB1−OPA1 interaction) in proteoliposomes, we used either 1 or 2.5 mM DSS (Thermo Fisher Scientific) for 30 min at room temperature. After cross-linking, the samples were quenched with 50 mM Tris-HCl (pH 7.4) for 15 min at room temperature and subjected to Western blotting with the indicated antibodies.

### Membrane fusion analysis

Membrane fusion analyses were performed using the previously established lipid mixing reaction (22) with some modifications (21, 37). A mixture of 2 μl of fluorescence-labeled liposomes (donor) and 5 μl of non-labeled liposomes (acceptor) was diluted with 11 μl of RB buffer and slotted into a 384-well black round-bottom microplate (Corning). In our condition, each donor and acceptor liposome contained 0.5 to 2 μM recombinant PHB1 and/or 0.5 μM L-OPA1 (0.25 and 1 μM were also used in the study) with 0.2 mM lipids, respectively. The reactants were pre-incubated for 10 min at 30° C in a fluorescence plate reader (Infinite F200 PRO; Tecan) and the fusion assay was initiated by adding 2 μl of GTP/MgCl_2_ at a final concentration of 3 mM or 2.5 mM, respectively. Membrane fusion reactions were monitored by the increase in the fluorescence intensity of NBD at 540 nm with excitation at 465 nm at 30° C. After reacting for 30 min, 2 μl of 4% (w/v) Triton X-100 was added to each sample to determine the absolute NBD fluorescence. The data were normalized to the percentage of total NBD fluorescence according to the following equation (22): lipid mixing (%) = (*F*_*t*_ − *F*_0_)/(*F*_*max*_ − *F*_*0*_) × 100, where *F*_*t*_ is the NBD fluorescence during the measurement, *F*_0_ is the initial NBD fluorescence immediately after the addition of GTP/MgCl_2_, and *F*_*max*_ is the NBD fluorescence after the addition of Triton X-100 to the reaction mixture. The first 2 min of each trace was fitted to a linear regression and the calculated slope was defined as the fusion activity.

### Negative-staining EM

A few microliters of proteoliposomes were applied to a glow discharge formvar/carbon-coated copper EM grid (PVF-C10–25, STEM Co, Tokyo, Japan) and the sample was allowed to settle on the carbon film for 5 min. The sample side of the grid was washed twice for a few seconds with a drop of water, and then the liquid on the grid was blotted off briefly and the sample side of the grid was immediately contacted with 1% (w/v) phosphotungstic acid (PTA) in distilled water. After 30 s, the excess PTA solution was blotted off with filter paper and the grids were air-dried. Images were captured with transmission EM (JEM-1400Flash, JEOL) at 80 kV.

### Confocal microscopy

HeLa cells were plated on glass-covered slips in 12-well plates (6 × 10^4^ cells per well). The following day, the cells were fixed with 4% (w/v) paraformaldehyde for 15 min at 37° C, permeabilized with 0.2% (w/v) Triton X-100 in 1 × phosphate-buffered saline (PBS, pH 7.2), and blocked with 5% (w/v) fetal bovine serum (Thermo Fisher Scientific). Mitochondria were visualized by staining with anti-CoxIV rabbit monoclonal antibody followed by Alexa Fluor 568-conjugated polyclonal antibody. For the RNA interference introduction experiment in HeLa cells, siRNA against human PHB1 (s10424) was purchased from Thermo Fisher Scientific. Cells were imaged with a C2 confocal microscope (Nikon Instruments Inc.).

### Statistical analysis

Statistical analysis was performed using GraphPad Prism 8.4. We considered different populations of cells to be biologic replicates; aliquoting or repeated measurements of a cell population was considered to represent technical replicates. We performed at least three independent reproducible results for most key experiments, although we did not perform explicit power calculations. Data are presented as the mean ± SD and statistical significance was assessed by one-way analysis of variance (ANOVA) followed by Tukey’s multiple comparisons or by two-way ANOVA followed by Bonferroni's multiple comparisons tests. A *p* value of less than 0.05 was considered statistically significant. We also used the chi-square tests with Bonferroni correction for comparing categorical data between groups.

## Data availability

All data are available contained in the main text and the supporting information.

## Supporting information

This article contains supporting information.

## Conflict of interest

The authors declare that they have no conflicts of interest with the contents of this article.
