# Supplementary material for: Prohibitin 1 tethers lipid membranes and regulates OPA1-mediated membrane fusion
Source: J Biol Chem. 2024 Dec 13;301(1):108076. doi: 10.1016/j.jbc.2024.108076 (PMC11760825; doi:10.1016/j.jbc.2024.108076)
Supplement: Supporting Information [file mmc1.pdf]

SUPPORTING INFORMATION

Prohibitin 1 tethers lipid membranes and regulates OPA1-mediated membrane fusion

Tadato Ban, Kimiya Kuroda, Mitsuhiro Nishigori, Keisuke Yamashita, Keisuke Ohta, and Takumi Koshiba

List of materials included:

Supplementary Fig. S1. Negative stain EM images of PHB1 reconstituted within MOM-mimicking phospholipids

Supplementary Fig. S2. Quantification of mitochondrial morphology in siPHB1-treated Hela cells

Supplementary Fig. S3. Cross-linking experiment of WT and its variant PHB1-IM

Supplementary Fig. S4. DLS profiles of PHB1-IM and PHB1^{I211P}-IM

Supplementary Fig. S5. Cross-linking experiment of OPA1 proteoliposomes

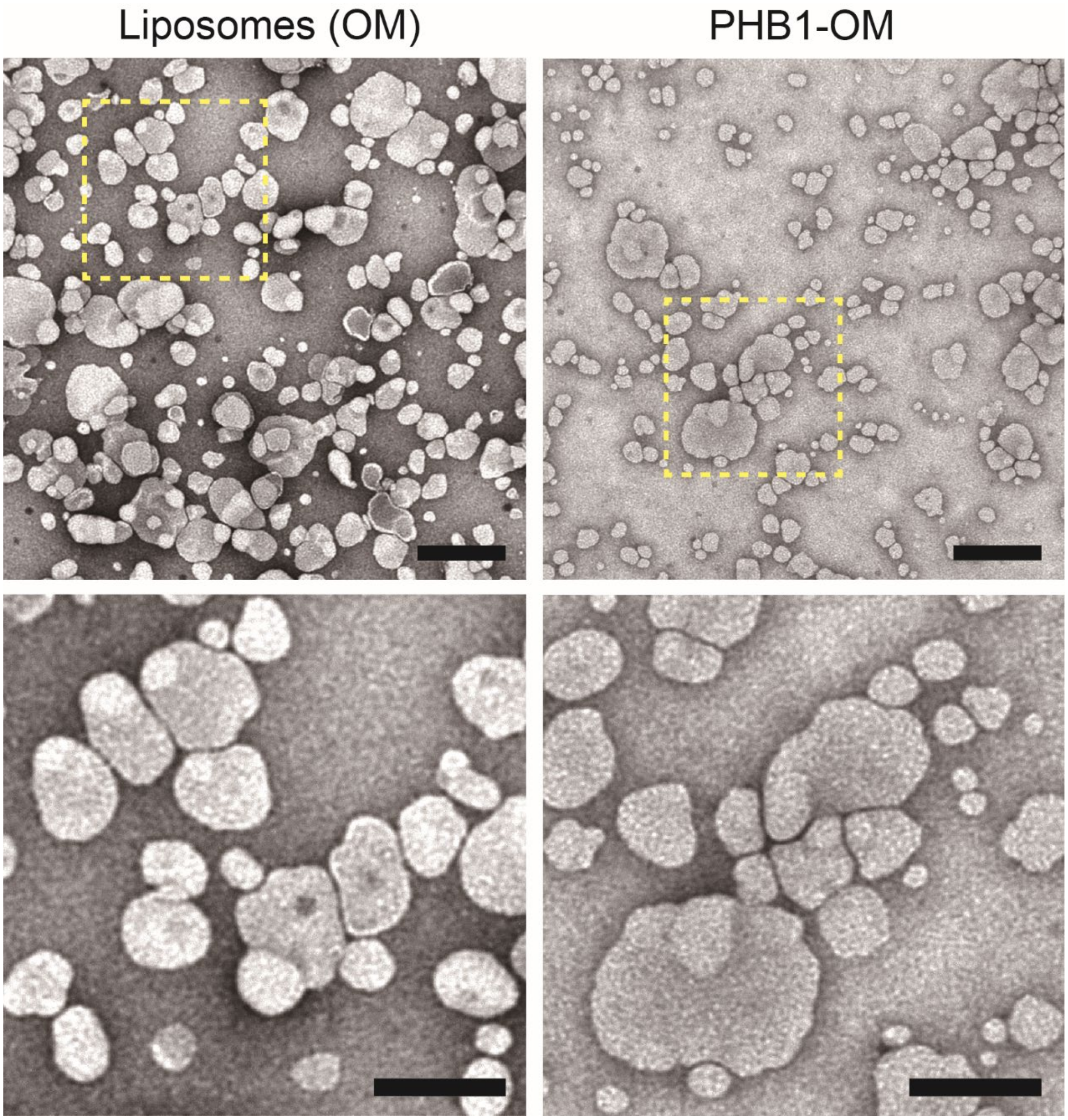

Supplementary Fig. S1 Negative stain EM images of PHB1 reconstituted within MOM-mimicking phospholipids (related to Fig. 2).

EM (negative staining) images of MOM-mimicking phospholipids (OM) and PHB1-embedded proteoliposomes (PHB1-OM). Bottom two images are magnifications of the yellow dashed areas in each top image. Scale bars, 200 nm (top) and 100 nm (bottom), respectively.

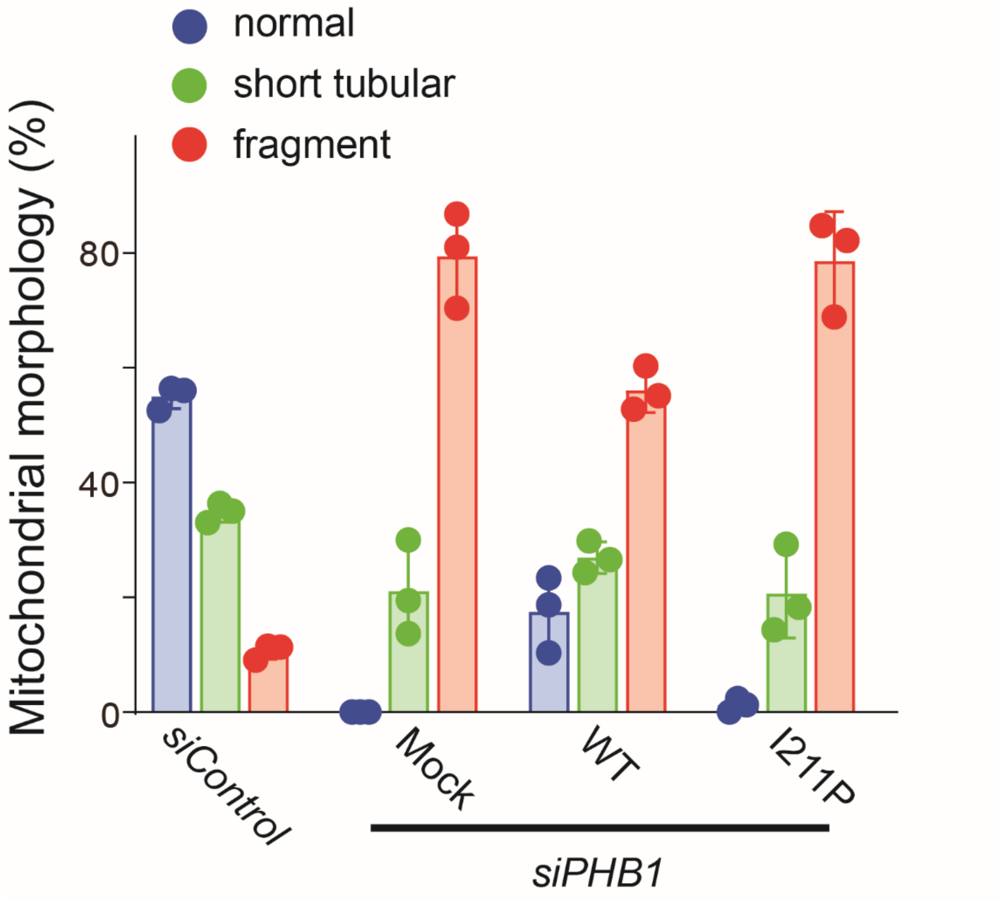

Supplementary Fig. S2 Quantification of mitochondrial morphology in siPHB1-treated Hela cells (related to Fig. 3).

The *siPHB1*-treated HeLa cells were transfected with a Myc-tagged version of either a WT or I211P PHB1 expression plasmid, and the mitochondrial morphology of the transfected cells (green) was monitored by immunofluorescence microscopy. Cells were classified into one of three morphologic categories as depicted (blue, green, and red), and at least 100 cells were assessed (*n* = 3).

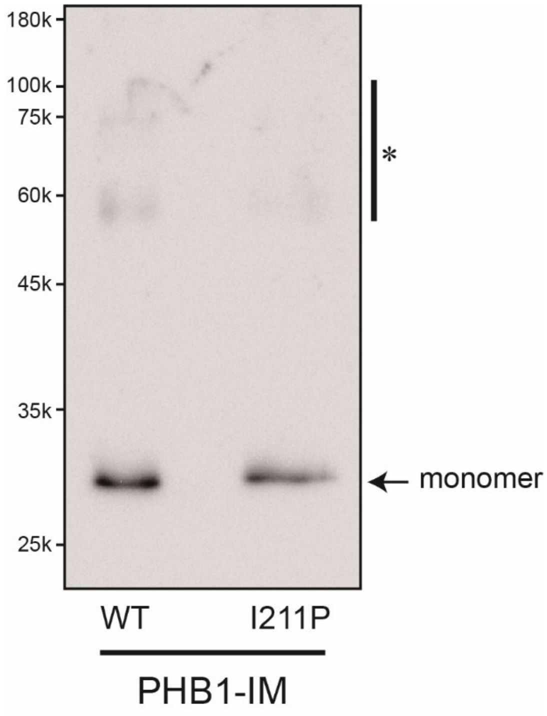

Supplementary Fig. S3 Cross-linking experiment of WT and its variant PHB1-IM (related to Fig. 3).

Chemical cross-linking experiment of PHB1-IM and PHB1^{I211P}-IM using DSS reagent (final concentration of 1 mM). Asterisk indicates homotypic PHB1 oligomerized bands and monomeric size of the protein is also shown.

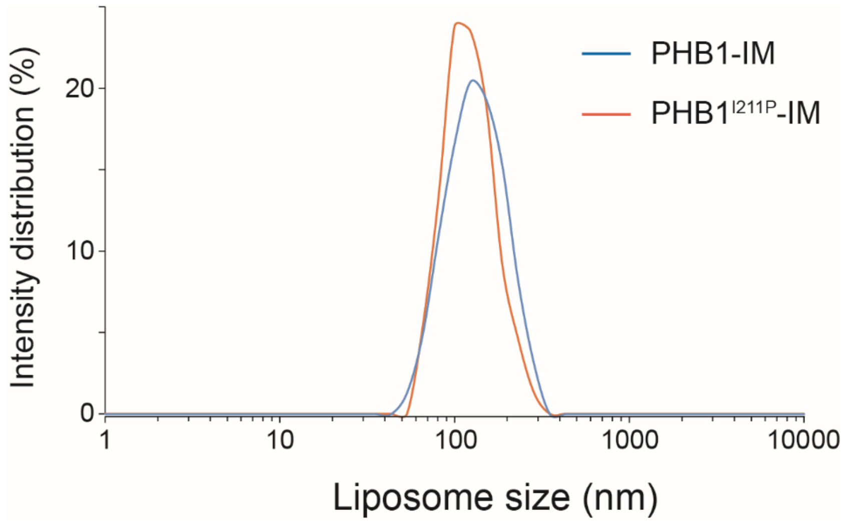

Supplementary Fig. S4 DLS profiles of PHB1-IM and PHB1^{I211P}-IM (related to Fig. 3).

The particle sizes of liposomes (IM) reconstituted PHB1 or PHB1^{I211P} were determined by DLS measurement at 25° C. Note that the particle size of PHB1-IM appears heterogeneous because of its broader distribution observed by DLS.

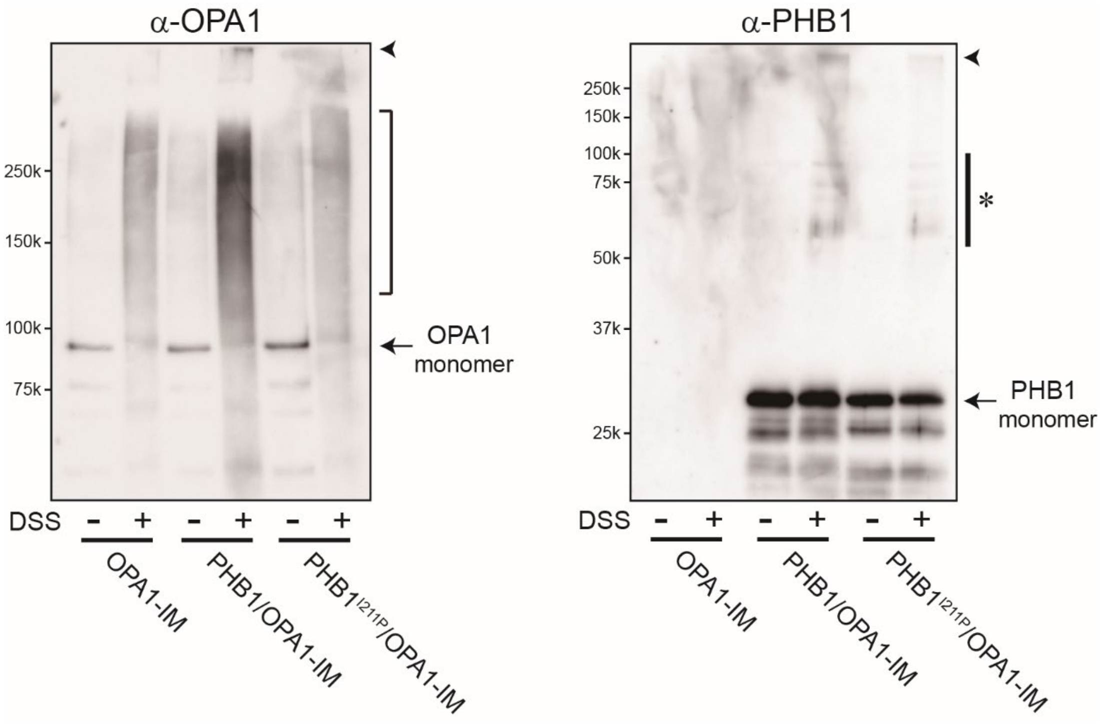

Supplementary Fig. S5 Cross-linking experiment of OPA1 proteoliposomes (related to Fig. 5).

Chemical cross-linking experiment of OPA1-IM in the absence or presence of WT or I211P PHB1 using DSS reagent (final concentration of 2.5 mM). Bracket in the left panel indicates cross-linkage OPA1 products and asterisk in the right panel indicates homotypic PHB1oligomerized bands. Higher cross-linkage products (arrowhead) were observed in PHB1/OPA1-IM.
